# Complex conversations in a healthcare setting: experiences from an interprofessional workshop on clinician-patient communication skills

**DOI:** 10.1186/s12909-021-02785-7

**Published:** 2021-06-14

**Authors:** Edward Stephens, Leeroy William, Lyn-Li Lim, Judy Allen, Bernadette Zappa, Evan Newnham, Kitty Vivekananda

**Affiliations:** 1 Eastern Health, Arnold Street, Box Hill, Victoria Australia; 2 Eastern Health Cancer Services, Eastern Health, Arnold Street, Box Hill, Victoria Australia; 3 Monash University, Level 1 Learning and Teaching Building, 19 Ancora Imparo Way, Clayton, Victoria Australia

**Keywords:** Communication, Interprofessional, Interdisciplinary, Skills, Learning

## Abstract

**Background:**

Communication is pivotal to the effective care and treatment of patients in our health care systems. Despite this understanding, clinicians are not sufficiently educated to confidently conduct complex discussions with patients. Communication skills workshops have been shown to be an effective educational format to improve clinician skills. However, despite the increasing interprofessional focus within modern medicine, there have been few studies looking at interprofessional communication workshops.

**Methods:**

A qualitative study was conducted to assess how an interprofessional communication skills workshop affected the communication skills of clinicians at a tertiary health service. Pre- and post-workshop surveys were undertaken by participants, followed by focus group interviews eight-weeks post workshop.

**Results:**

Clinicians were able to incorporate learnt communication skills into their daily practice. This was associated with an improvement in confidence of clinicians in having complex discussions, in addition to a reduction in the burden of having complex discussions. Participants responded positively to the interdisciplinary format, reporting benefits from the learning experience that translated into daily practice.

**Conclusion:**

Clinicians’ communication skills in conducting complex clinician-patient conversations can be improved by participation in interprofessional communication skills workshops. We identified that the interprofessional aspect of the workshops not only improved interprofessional understanding and relationships, but also developed increased self-awareness during complex discussions, and reduced the sense of burden felt by clinicians.

**Supplementary Information:**

The online version contains supplementary material available at 10.1186/s12909-021-02785-7.

## Background

Excellent communication between clinicians and their patients is essential for effective, safe, and patient-centered healthcare [1–3]. Any consultation between a clinician and patient is overlaid with emotions and differing expectations. This can cause confusion between what is spoken and what is actually understood by both parties. For patients with chronic and complex medical conditions, where emotive discussions may involve treatment goals and end-of-life discussions, clear and compassionate communication is even more challenging. It has been recognised that doctors may not have the communication skills or confidence to have these complex discussions [4–6]. It is unsurprising therefore, that these conversations are not occurring [7, 8], despite evidence suggesting that patients want to have these discussions with their treating clinicians [9].

Optimal healthcare delivery depends upon excellent communication by the healthcare professional. The academic literature is limited on interprofessional communication skills education, although evidence exists that it can improve communication with patients effectively and efficiently [10, 11].

The deficiency in communication skills education is well-recognised by universities and teaching hospitals, leading to a lack of systematic education [1, 12].. Evaluations of different delivery approaches including lectures, computer-based modules, and video/audio review of communication skills [13], suggest that ‘communication skills workshop’ approach is superior to others [14–18].

The multidisciplinary nature of the vast majority of communication in medicine is increasingly recognised [2]. In recent years, there has been more research involving nurses and doctors in interprofessional communication skills education [19–21].

Our study assessed how an interprofessional communication skills workshop affected the communication skills of clinicians, and how the interprofessional nature affected their experiences.

## Methods

### Aim

To evaluate how a single interdisciplinary communication skills workshop affected the communication skills of participants. We additionally evaluated the role of the interdisciplinary component on the workshop’s outcomes.

### Design

We evaluated the experiences of participants using focus group interviews six-weeks after participating in a single interprofessional communication skills workshop. Pre and post-workshop surveys were undertaken by participants, followed by focus group interviews six-weeks post workshop.

### Setting

This was undertaken at a single tertiary health service.

### Participants

Expressions of interest were sought from the health service clinician groups identified as regularly required to conduct complex clinician-patient conversations. Invitations were sent to forty registrars from general medical and emergency training programs (with availability of these registrars for in-hours training) as well as twenty nursing and allied health staff. Invitations with information regarding the communication workshop, surveys and focus group sessions were e-mailed to participants. The participant information form explained the role of the communication workshop, however did not give information about the scenario or focus on end of life conversations. A convenience sample of twenty participants were involved in responding to the pre- and post-workshop survey and ten participants attended the two focus group interviews. All twenty participants attended a single communication workshop. Participants were admitted into the study from their order of response to the advertisement. No randomisation of participants took place. Consent to participate in the study was implied by completion of the pre-workshop survey.

### Pre-workshop survey

The study consisted of an anonymous pre-workshop survey (distributed by e-mail via a SurveyMonkey® link) that identified prior experiences, current attitudes to communication and goals of participation. Responses to both pre and post surveys were made in the form of open answers, multiple choice or by Likert scales (see appendices). Survey questions were adapted by the researchers from previous unpublished surveys conducted at the health service prior to commencement of the study.

### Communication skills workshop

Scenarios based on end-of-life conversations were used given their significant clinic and emotional complexity.

Each participant attended a single four-hour workshop with 5 participants per workshop. Three experienced facilitators from medical [11] and allied health backgrounds (JA, BZ) conducted the workshops. A trained actor with prior experience in simulated patient education was used to facilitate interactive communication skills training. The workshop consisted of a brief period of didactic communication skills education focused on identifying key communication tools. This was followed by individualised interactive role-plays. Each participant performed a different component of a clinician-patient interview, utilising specific communications skills pertinent to their role-play. The audience provided commentary on the interaction, the cues noted, and the skills identified or utilised during the role-play. Facilitators provided guidance and feedback regarding the consultation during and following each role-play.

### Post-workshop survey

An anonymous post-workshop survey (SurveyMonkey®) was distributed via e-mail 6 weeks following the workshop. Responses were not linked to the pre-workshop survey. This survey aimed to ascertain use of particular communication skills and changes in confidence during end-of-life care discussions following the workshop. It also sought to review if the workshop had changed the identification of patient’s requiring goals of care discussions and the participant’s engagement with these discussions.

### Post-workshop focus group interviews

Participants were invited to a one-hour semi-structured focus group interview 8 weeks following completion of the workshops. Participation was voluntary. Two focus groups were held, with participants capped at five per session. Ten (50%) of the workshop participants (five medical staff, four allied health and one nursing) attended focus group sessions. Non-attendance reflected lack of availability due to work-related commitments.

In order to mitigate against bias, the focus group sessions were run by an external psychologist and researcher (ES) who had no pre-existing relationship with participants. A brief semi-structured interview guide was developed for this study by ES, KV (see supplementary material) with open-ended questions to explore the impact of the communication workshop, and to identify key benefits and challenges to interprofessional communication during end-of-life care conversations. Due to time constraints the interview questions were not pilot tested.

#### Analysis

##### Quantitative analysis

Descriptive analysis was undertaken of the pre- and post-workshop surveys.

##### Qualitative analysis

Verbatim written transcriptions were made from the audio recordings of the two focus group discussion. Qualitative analysis was undertaken using a multidisciplinary team (ES, LL, LW, KV). Two arms of qualitative research were able to be carried out. Deductive analysis was obtained through responses to questions in the surveys. Inductive analysis was completed through the use of the focus group sessions.

Using Lundman & Graneheim’s steps for qualitative content analysis of transcripts, meaning units were identified, labelled and condensed [22]. To ensure credibility and trustworthiness of the qualitative analysis, underlying meaning, new descriptive themes and subthemes were identified, categorised, cross-checked and verified independently by two researchers (ES, LL) until inductive thematic saturation was reached [20, 21].

## Results

### Quantitative results

The demographic analysis of the twenty participants is presented in Table 1;90% of participants had more than 3 years clinical experience. Less than half of the participants had previously attended a communication skill workshop. Participants were surveyed on whether previous exposures were felt to be helpful in their clinical practice. Of those who attended the workshop, 35% (*n* = 7/20) had previously attended a communication skills workshop during their undergraduate (pre-clinical) training. Of these 71% (*n* = 5/7), found it helpful in later clinical practice. Only 20% (*n* = 4/20) of the participants had attended postgraduate training via communication skills workshops, of which 100% (n = 4/4) found these beneficial to their clinical practice. Of note, the four who had attended postgraduate education had also attended undergraduate communication skills education.

**Table 1:** Demographics and key respondent data to the pre-workshop survey

Medical		***n*** = 20	%
	Basic Physician Trainees	9	45%
	Advanced Physician Trainees	3	15%
	Emergency Physician Trainees	1	5%
**Nursing**	Intensive care liaison nurse	1	5%
**Allied Health**	Physiotherapists	2	10%
	Occupational Therapist	1	5%
	Dieticians	3	15%
**Prior communication skills workshop attendances**			
	Undergraduate	7	35%
	postgraduate	4	20%
	no previous comm skills teaching	13	65%
**What have you found most valuable in generating your method for discussion of goals of care?**			
	observation	17	85%
	experience	15	75%
	communication skill workshop	7	35%
	lectures	2	10%
	computer based education	0	0%
	other	0	0%
**What are the barriers to having goals of care discussions?**			
	correct use of language	15	75%
	risk of creating conflict	14	70%
	lack of adequate knowledge	13	65%
	time	11	55%
	lack of adequate confidence	10	50%
	management of cultural belief	10	50%
	environment	7	35%
	patient/family acceptance of illness	4	20%

All twenty participants completed the pre- and post-workshop surveys. Participants were surveyed on the importance of goals of care discussions in their clinical practice. Of the twenty respondents, nineteen identified goals of care discussions as important or very important (95%) on a Likert scale.

Results from the post-workshop survey identified the most common communication skills utilised by participants were: the use of silence (*n* = 9/20, 45%), summarising (*n* = 5/20, 25%), and signposting (*n* = 3/20, 15%). All participants reported that the workshop was useful in providing skills for goals of care discussions, with 90% noting interest in future communication skills workshops.

### Qualitative results

Thematic analysis from the focus groups provided two key themes in relation to the experience of complex end of life care conversations. Firstly, the development of communication skills and secondly the theme of interprofessional relationships. Within the development of communication skills, four subthemes were elucidated: increased confidence; increased self-awareness and reflective practice; rapport and relationship building; reduced burden and the impact that this had on the clinician.

When analysing responses to the educational workshop two key themes were identified: the safety in teaching and the role of the interdisciplinary format. The safety in teaching theme also had a subtheme of vulnerability and its effect on the clinicians.

### Experience of complex end-of-life care conversations

#### Communication skills

The use of key communication tools was identified by participants as helpful in their routine communication. Focus group participants explained how their daily practice had changed following the use of skills such as silence.

[use of silence] *“I’ve heard the patients say a lot more. I’ve found out more with saying less. It has been important to hear what they have to say, and work it out for themselves.” (Physiotherapist 1, male)*

In addition to the use of silence, summarising was identified by workshop participants as useful in their practice. Summarising in particular was seen as a clear way of building rapport and efficiency establishing shared goals.

[using summarising] “*I can build rapport much quicker, and end up being able to provide the therapy that I need to provide in a quicker manner and with better results.” (Occupational Therapist 1, female)*

Allied health clinicians had particularly noted that engaging in active empathy and naming emotions had improved the brevity of their communication. This had allowed for a more indepth understanding of their patient’s needs.

*“I think I used to fluster myself tyring to be sympathetic. Now I can hear what is being said to me and say that emotion they are feeling is valid rather than saying ‘that is unfortunate how about we try this’ or ‘yes, I understand but let’s try this’. I don’t feel I have to give a solution.” (Dietician 2, female)*

#### Confidence

*Before, the goals of care discussions scared me … whereas now I am more confident in having that discussion … . For example, before, a patient who was at the end of life, I wouldn’t see early because I would let the medical team and social worker go in first. Now though, I feel more confident in going in and having the conversation (Dietician 1, female)*

The theme of confidence was present in all medical, nursing and allied health disciplines responses. Given the strength of this theme it was explored further in terms of how it had altered or impacted daily communication. Respondents were able to describe ways in which they recognised times of poor communication and corrected themselves.

*“I am also aware of, if I don’t say something clearly, or how I would like to, I can acknowledge that and put it in a different way” (Doctor 3, female)*

Exploration around the theme of confidence in communication led to discussions regarding effects on the provision of patient-centred care. The outcome described by participants during the focus group sessions were of an improvement in their ability to perform patient-centred care. In particular, allied health clinicians identified the ability to more accurately identify focal areas of their patient’s concern and enact appropriate therapeutic interventions.

*“I can identify the patient’s priority more, and identify what I need to do. Sometimes after the discussion I find that I can help them less from a physiotherapy perspective, but that is ok because it is not the patient’s priority.” (Physiotherapist 1, male)*

#### Self awareness and reflective practice

A key feature raised by multiple participants at the focus group was the role of increased self-awareness. Participants recognised the importance of actively engaging in reflective practice around communication. This was mentioned across all disciplines with particular recognition by junior doctors.

*“I am more aware of what I was doing … I had never been aware like that before. I usually get caught up in the structure of the conversation and trying to work within that structure to the next phase but I thought, well I’m doing things a bit differently now” (Doctor 2, male)*

Following the communication workshop participants displayed the ability to develop reflective practice during communication. This enabled the participant to utilise the most effective communication skill in a particular setting.

*“So, you have become a very conscious communicator?” (Interviewer)*

*“I have thought about it more when I have been struggling with a discussion, or not known where I was heading. I have used the teaching more. I think you become more aware. (Dietician 1, female)*

#### Rapport and relationships: clinician-patient

A consistent consequence of increased confidence noted by the participants was how this was positively affecting the patient-physician relationship and ability to build rapport.

*“having that confidence helps build more rapport because then I am able to focus more on the picture that I am seeing rather than focusing on what I am saying … ” (Doctor 1, male)*

Participant responses allowed for the recognition of the strong theme of relationship building. In both focus group sessions, the participants noted that their clinician-patient relationships had been improved by use of skills learnt during the communication workshop

*“I can build rapport much more quickly and end up being able to provide the therapy that I need to provide in a quicker manner and with better results”. (Physiotherapist 1, male)*

#### Reduced burden

A reduction in the burden felt by hospital staff in having complex goals of care discussions was noted in the focus group session. In particular the allied health participants acknowledged that their communication skills training had allowed them to engage with their patients’ needs, rather than their own objectives.

*“I was always trying to find an answer whereas now I understand that it is all right not to say anything then I don’t have a solution and knowing that it is ok to not have an answer … I’m more comfortable not saying something rather than blurting something out just to fill the gap” (Dietician 2, female).*

#### Interprofessional relationships

All participants reported an improved recognition of each other’s disciplines, and their role and experience in communication. All participants identified different aspects of other attendee’s communication that they had learnt from the communication workshop.

*“I thought it was really helpful to have the different approaches. Doctors have a different approach to allied health and it is nicer to see that broader approach … ” (Doctor 1, male).*

Participants identified that not only had patient-clinician relationships altered following the communication workshop, but also clinician-clinician relationships. Participants were able to identify colleague’s individual skills and utilise these.

*“I think that good communication enhances the overall team, and I’ve found that* [since the workshop] *I’m being asked what I would do with a particular patient. It’s more of a two-way street now which is lovely” (Nurse 1, female).*

### Experience of the workshop

#### Safety in teaching

The format of the interactive communication skills workshop was identified by all participants as initially disconcerting. Participants found performing in front of colleagues and facilitators challenging. However, they also identified this challenge as a key part of the learning process.

*“It is because it does put you out of your comfort zone so much that it is so effective. It wasn’t just sitting and listening” (Dietician 3, female)*

Discussion during the focus group identified that despite this initial discomfort, all participants engaged well with the process.

*“I think the environment we did it in made you feel comfortable. I was nervous but it was good that we could individualise it, take breaks and practice a particular skill.” (Dietician 2, female)*

#### Vulnerability

All disciplines discussed the role of needing to engage with some level of vulnerability in order to get the most out of the workshop.

*“ … I was nervous but it was good that we could individualise it … I was the only allied health professional in our session so I felt a bit vulnerable, but actually when I went, I felt comfortable.” (Dietician 2, female)*

#### Interprofessional relationships

Participants identified that completing the session with colleagues outside of their own sphere of practice was beneficial. This was not just from a skills development perspective, but also from a health service provision perspective.

*“It gave me a realistic impression of the discussion that the medical staff need to have and how difficult that can be. It also brings the team together. I think it will improve our team work to have the same training in communication.” (Occupational therapist 1, female)*

All participants in the focus group sessions found the communication skills workshop effective, well run and expressed an interest in future sessions. When asked regarding opportunities for improvement, clinicians expressed an interest in recordings of the workshop to provide audio-visual prompts for future use and revision following the workshop.

## Discussion

This study supports the positive influence of a single interdisciplinary communication skills workshop on the communication skills of medical, nursing and allied health clinicians over a two-month period. Our results build the growing evidence for an interprofessional focus on education in the healthcare sector [18, 23].

Our study demonstrated that participants’ experience of conducting complex clinician-patient conversations were improved by participation in an interprofessional communication skills workshops where they were provided the opportunity to observe and practise practical communication strategies.

Prior to the workshop, participants self-identified significant concerns in their own communication skills. Correct use of language was identified by the majority of respondents as their key barrier to communicating with their patients. Risk of causing conflict also featured as a significant concern for respondents. Following the workshop, participants identified that they utilised learnt communication skills to manage complex discussions. Key features identified by participants were, the ability to engage actively with the communication process. This allowed participants to generate meaningful discussion with patients, exploring patient’s goals and concerns, rather than focusing on the interviewer’s objectives. This alleviated concern regarding conflict, and, evidenced an improvement in clinician-patient relationships. This is supportive of the current evidence [23, 24].

This study confirms that clinicians lack confidence in carrying out complex discussions. This is in line with current experience in literature [4, 5, 25]. Our qualitative data suggests that participants experienced an improvement in their confidence during discussions following the communication workshop. The themes of increased self-awareness and reflective practice were identified as adaptive self-assessment mechanisms by participants during focus group discussion. This suggests that their pre-workshop self-identified confidence may have been an optimistic appraisal. This highlights the challenges of self-assessment particular of ‘confidence’. Furthermore, engagement in the workshop provided participants with insight into the challenges of communication and assessment of their own communication skills. When faced by challenging discussions in their daily practice following the communication skills workshop, participants had the confidence to use learnt skills to manage their communication.

We also explored participants’ experiences with the workshop and mode of delivery, in particular its interactive approach. Individual participants engaged well with the communication skills workshop activity. Combined qualitative and quantitative data, including focus group responses around vulnerability and safety in teaching, suggest that clinicians are willing to actively engage with communication skills training. Our findings build on previous literature [23, 26] that, despite the uncomfortable nature of interactive communication skills training, with professional facilitation, the process can provide a meaningful learning experience. Focus group responses suggest that the perceived discomfort and ‘vulnerability’ were the pivotal factors that consolidated engagement with the learning process. All participants found the sessions helpful in improving their communication skills in daily clinical practice. Participants expressed a clear interest in future engagement with a similar program. In particular, this study suggests that involving medical and allied health disciplines improves interdisciplinary understanding and communication skills [1, 25, 27].

Participants reported that utilising an interprofessional educational environment improved their experience of undertaking patient-centred clinician-patient discussions. Participants within the focus group clearly identified benefits of conducting the workshop with a broad range of clinicians. This diversity of participants’ perspectives in response to workshop scenarios was identified as a key learning point in focus group responses, and later utilised in clinical practice by the different disciplines. Participants reported a self- assessed improvement in their communication skills in practice, both with patients and also interprofessional team members., and ability to address issues with their patients outside of their usual scope of practice. Furthermore, there was a perception that the change in practice did not compromise the patient-clinician relationship within the team, but in fact appeared to strengthen it.

Conducting the focus group sessions and subsequent analysis raised the theme of improved interprofessional relationships. In a period of fast-paced interprofessional medicine, disciplines are often increasingly siloed [28]. Participants identified a strong sense of shared responsibility following the communication workshop. Overall, participants remarked that this had improved their overall satisfaction at work. This adds to previous literature identifying that good communication not only improves job satisfaction but also rates of burnout [1, 29, 30].

It is important to recognise that this study assessed the subjective experience of changes in participant’s communication skills and confidence. The subjective change in confidence does not however, necessarily reflect an improvement in overall competency [31]. The optimal method for assessment of competency remains elusive and is not within the scope of this study [32]. Furthermore, the limited number of participants provides further challenges in analysing behavioural change. However, our focus on identification and utilisation of particular skills and the context of this in our focus group responses provides evidence towards communication practice modifications.

There were a number of limitations to this study. From a methodological perspective, the qualitative analysis was carried out by the research team, leading to the possibility of a biased thematic analysis. To try to address this bias we utilised a broad range of researchers in our analysis from both clinical (both medical and allied health) and non-clinical fields. This study had a small sample size of twenty participants in the surveys and ten in the focus groups which limits generalisability. Furthermore, participation was voluntary leading to a possible selection bias of participants who were already interested and proactive in improving their communication skills education. In order to address these issues, a service-driven policy advocating communication skills education with departmental commitment to professional development would be required. This study provides some early data about the benefits of such a strategy. A further limitation arose from participant comprehension of ‘goals of care’, ‘end of life care’ and ‘resuscitation’ workshop discussions. It was identified by workshop facilitators that there was a large variability in the understanding of ‘goals of care’. Thus, after education at the workshop the follow-up survey responses were likely to more accurately reflect practice.

This study provides the basis for further development of interprofessional communication workshops and research into their efficacy. Future research would benefit from larger cohorts, and the use of serial communication workshops to consolidate and build on communications skills. Additionally, the use of video recordings of clinician–actor interactions could help further analysis of communication skills for participating clinicians.

## Conclusion

This study demonstrates that a single interactive, interprofessional communication skills workshop can help to improve clinician communication skills when undertaking complex clinician-patient discussions by improving confidence and introducing skills that could be incorporated into practice.

As well, it highlights the potential for interprofessional learning in communication skills, as well as the willingness of clinicians to engage in structured workshops. Ultimately, healthcare providers have a duty to improve communication on many levels to benefit patients, families, and staff. Although communication remains the biggest source of complaints [33] in the health sector, we believe that a systematic approach to increased interprofessional communication skills training would be of generalised benefit.

## Supplementary Information

**Additional file 1.**

**Additional file 2.**

## Data Availability

The datasets used and analysed during the current study are available from the corresponding author on reasonable request.
