# Supplementary material for: Complex conversations in a healthcare setting: experiences from an interprofessional workshop on clinician-patient communication skills
Source: BMC Med Educ. 2021 Jun 14;21:343. doi: 10.1186/s12909-021-02785-7 (PMC8204413; doi:10.1186/s12909-021-02785-7)
Supplement: Supplementary file 1 — Additional file 1. [file 12909_2021_2785_MOESM1_ESM.docx]

**Pre- communication skills workshop survey:**

| 1 | What is your gender? M/F |
| --- | --- |
| 2 | How many years of clinical practice have you undertaken? |
| 3 | What is your current area of training?  HMO/BPT/AT/Allied Health – discipline |
| 4 | How important would you rate ‘Goals of Care Discussions’ in your daily practice? |
| 5 | How many ‘Goals of Care discussions’ would you have in a normal working week? |
| 6 | How would you rate your confidence during these discussions?  (Not confident / mildly confident / confident / very confident) |
| 7 | What do you see as the key barriers to good communication in your daily practice?  Time / language / cultural beliefs / ward set-up / knowledge / confidence / family vs treating team expectations / open response / patient acceptance of illness / death / prognosis |
| 8 | Have you previously attended a communication workshop and at that time was it helpful?  Undergraduate y/n  Looking back do you think that it was helpful y/n  Postgraduate y/n  Looking back do you think that it was helpful y/n |
| 9 | What have you found most valuable in generating your method for conducting end of life care discussions (eg: direct observation, comm skills training, on-the-ground experience, other) |

**Post- workshop survey:**

| 1 | What is your gender? M/F |
| --- | --- |
| 2 | How many years of clinical practice have you undertaken? |
| 3 | What is your current area of training?  HMO/BPT/AT/Allied Health – discipline |
| 4 | Following your participation in the communication workshop how would you now rate your confidence during goals of care discussions?  (Not confident / mildly confident / confident / very confident) |
| 8 | Did attending the communication workshop provide you with useful tools to assist your ability to perform discussions in the clinical setting?  Yes/no  Open answer |
| 9 | What communication tool have you found most useful or utilised most frequently following the communication workshop?  Silence / non-verbal communication / summarizing / chunking and checking / sign posting / active empathy |
| 10 | If you had further opportunity to attend communication training would you want to participate?  Yes/no |
| 11 | If further communication workshops are offered are there specific areas of communication that you would be interested to develop? i.e. general communication techniques, communication with families, breaking bad news, resuscitation discussions, end of life care, goals of care discussions. |
| 12 | What barriers would prevent you from attending a communication workshop? |
| 13 | What alternative formats of education would you engage with? I.e. ilearn / paper based / video tutorials / ward-based teaching |
