# Supplementary material for: Complex conversations in a healthcare setting: experiences from an interprofessional workshop on clinician-patient communication skills
Source: BMC Med Educ. 2021 Jun 14;21:343. doi: 10.1186/s12909-021-02785-7 (PMC8204413; doi:10.1186/s12909-021-02785-7)
Supplement: Supplementary file 2 — Additional file 2. [file 12909_2021_2785_MOESM2_ESM.docx]

Complex conversations in a healthcare setting: Experiences from an interprofessional workshop on clinician-patient communication skills

Semi-structured focus group stimulation questions:

1) What about the training has been helpful in having complex and goals of care conversations? How have you been able to use it? what have you been able to implement?

2) What barriers exist for having complex and goals of care conversations?

3) What would you change about the training? or future training needs
